# Editorial: The Emergent Engram: Multilevel Memory Trace Components and the Broader Interactions

**DOI:** 10.3389/fnbeh.2022.929248

**Published:** 2022-05-27

**Authors:** Bryan D. Devan, Robert J. McDonald

**Affiliations:** ^1^Laboratory of Comparative Neuropsychology, Psychology Department, Towson, MD, United States; ^2^Department of Neuroscience, University of Lethbridge, Lethbridge, Canadian Centre for Behavioural Neuroscience, AB, Canada

**Keywords:** theory, constructs, engram, cell assembly, memory trace

In this Research Topic, a follow up to Devan et al. (2018), a diversity of contributions were included, fulfilling the primary objective of the topic: to provide a collection of diverse research and ideas on the *emergent engram* – an integration of mnemonic processes at multiple levels of organization: molecular, epigenetic, cellular, systems and circuits, using different neuroscience techniques and psychological approaches. The diversity of contributions include: *Original Research* on episodic memory reconsolidation and whether the intention to encode influences older reactivated memory traces (Simon et al.); the role of the mPFC and the vHPC in the spatial retrieval of a previously learned active place avoidance (Cernotova et al.); targeted memory reactivation to improve memory consolidation when re-applied during sleep (Beijamini et al.); and hippocampal competition with other memory systems (overshadowing) on visual discrimination dependent on the hippocampus (Lehmann et al.).

The Research Topic also included important *Review Articles* on place and response learning studies, focusing on the historical foundations and neurobiological findings (Goodman); the historical contributions of William McDougall's theory of synaptic change that pre-dated the “Hebb synapse” (Brown et al.); and assumptions about engrams and the important role of interneurons in synaptic plasticity during different state-regulated memory processes (Raven and Anton).

## The Broader Implications

Related to the above articles, and to the broader literature, there is a conceptual issue on the precise use of terms that should be clarified. Semon created his own terms to avoid potentially misleading connotations of everyday language related to memory. Precision of terminology is fundamental to the relation between theoretical constructs in science and operationalism of valid measurement (Agassi, 1968).

Semon (1904, 1909, 1921, 1923) coined one of the best-known terms in neuropsychology, the “engram” (Schacter, 1982, 2001), derived from the Greek, “that which is written in” (Glickstein, 2014; p. 241). It has been compared to Donald Hebb's “cell assembly” (Milner, 1989) and a “memory trace,” originally of philosophical origin (e.g., Sutton, 1998), that is now prominent in neuropsychology (De Brigard, 2014).

## Comparison of Definitions

1. **Engram**– “…the enduring though primarily latent modification in the irritable substance produced by a stimulus” (Semon, 1921, p. 12).
2. **Cell assembly**– “…a diffuse structure comprising cells in the cortex and diencephalon (and also, perhaps in the basal ganglia of the cerebrum), capable of acting briefly as a closed system, delivering facilitation to other such systems and usually having a specific motor facilitation (Hebb, 1949; p. xix).
3. **Memory trace**– “…the modification of the anatomical substrata of fibers and cells, or of the physiological activity, which is the occasion of …the reproduced idea” (Maudsley, 1878, p. 513). Memory traces were “…posited as existing in the brain, and as persisting for a particular length of time” (James, 1890; p. 655).

## Semon's Engram

Semon's definition of the engram is often used synonymously with the word “memory trace,” which specifically applies to the brain. However, in *The Mneme*, Semon (1921) defined the engram to include the process of heredity (e.g., Dendy, 1912). In fact, Semon believed that heredity and memory were the same phenomena (organic memory) a position that he adopted as a neo-Lamarckian evolutionary theorist (Schacter et al., 1978; Schacter, 1982, 2001). Do modern neuroscientists base the resurgence of the term engram on its dual application to memory as “inheritance by acquired characteristics” the same as the brain processes involved in memory formation? Semon did, using the commonly cited definition above (28 verbatim citations on Google Scholar). Further, contrary to Semon's intent, the term is now part of everyday language (Devan et al., 2017).

Semon's second book (Semon, 1909, 1923), *Mnemic Psychology*, focused on engrams in the brain after experimental genetics at the time seemed to refrute neo-Lamarckian inheritance and organic memory. Simon experienced several personal tragedies at this time in his life and his “close friend” (see Schacter, 2001; p. 180, for discussion) Vernon Lee wrote a 42-page introduction to Semon's theory, even changing the title of the book from Semon's German “mnemic sensation” (and suppressing its subtitle) to Vernon's title of “mnemic psychology” (Semon, 1923, p. 11).

## Hebb's Cell Assembly

Of the three related terms above, the cell assembly and the memory trace are specific theoretical constructs of brain function related to cognition and behavior. For the cell assembly, notice that there is no inherent memory function. In fact, cell assemblies often represent stimuli and perceptions, the precursors of memory formation. It is only when cell-assemblies undergo “synaptic change” forming “reverberating circuits” of continuous excitation that outlast the stimulus do they then take on the function of short-term memory. Sets of cell-assemblies may then form a “phase sequence,” which Hebb described as the “thought process.”

A student of Hebb's, Peter Milner, states: “Hebb postulated distributed memory traces consisting of lattices of neurons linked together during learning by increased effectiveness of their mutual synaptic connections” (Milner, 1989, p. 24). Obviously, there is a considerable relationship between the engram, memory trace and cell assembly, although as many authors point out, they are only “roughly” the same. While the definition of the engram was broadly conceived to unify heredity and memory, the memory trace and the precursor cell assembly are specific brain constructs. It seems curious that many contemporary neuroscientists seem to prefer the term engram to the more specific alternatives.

**Figure 1:**
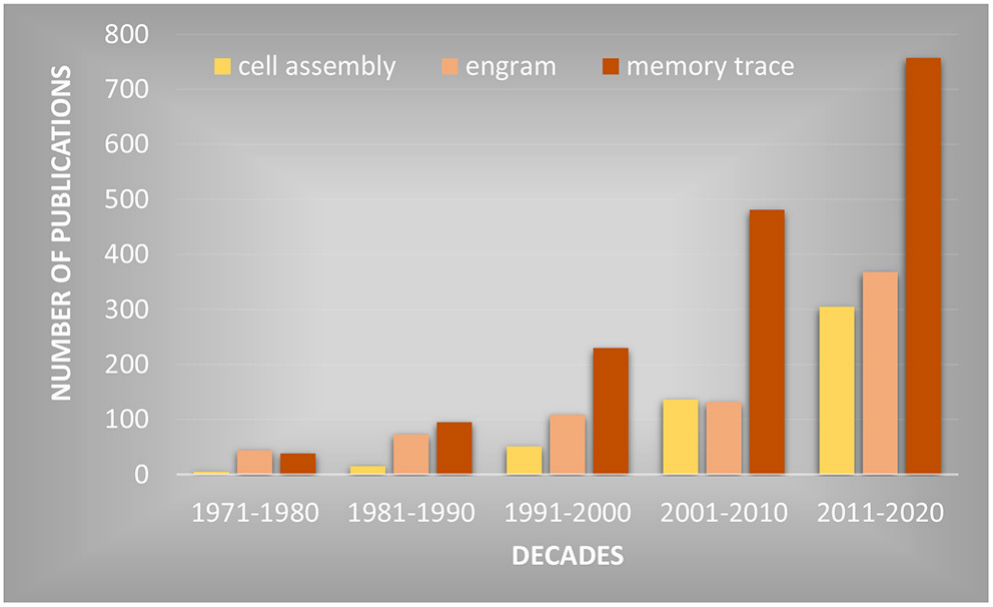
The number of publications retrieved from the PubMed database that use the terms “Engram,” “Cell Assembly” and “Memory Trace” across the past five decades shown on the vertical axis.

Some researchers have claimed that technological advances in neuroscience (e.g., optogenetics) have led to the discovery of the engram. Despite the correspondence, the term memory trace is used significantly more in the biomedical literature based on several database searches. A PubMed database search is shown in Figure 1. Clearly, the term memory trace has gained far greater usage in recent decades than the alternatives. Hebb's cell assembly uniquely applies to brain representations of sensory-perceptual processing networks that are widely distributed throughout cortex (Lashley, 1950) and subcortical systems (Thompson, 1983; Thompson et al., 1990). When closed circuit reverberation occurs, synaptic plasticity forms memory traces. In contemporary usage, the engram seems to apply specifically to the brain, however its history is one that is contrary to what most neuroscientists would agree, that memory is not heredity, but a separate process of brain function.

## Author Contributions

BD and RM contributed to the conception of the Research Topic. BD wrote the first draft of the manuscript. RM assisted with reviewing the manuscript. All authors contributed to manuscript revision, read, and approved the submitted version.

## Conflict of Interest

The authors declare that the research was conducted in the absence of any commercial or financial relationships that could be construed as a potential conflict of interest.

## Publisher's Note

All claims expressed in this article are solely those of the authors and do not necessarily represent those of their affiliated organizations, or those of the publisher, the editors and the reviewers. Any product that may be evaluated in this article, or claim that may be made by its manufacturer, is not guaranteed or endorsed by the publisher.
